# *Kcne2* deletion causes early-onset nonalcoholic fatty liver disease via iron deficiency anemia

**DOI:** 10.1038/srep23118

**Published:** 2016-03-17

**Authors:** Soo Min Lee, Dara Nguyen, Marie Anand, Ritu Kant, Clemens Köhncke, Ulrike Lisewski, Torsten K. Roepke, Zhaoyang Hu, Geoffrey W. Abbott

**Affiliations:** 1 Bioelectricity Laboratory, Dept. of Pharmacology and Dept. of Physiology and Biophysics, School of Medicine, University of California, Irvine, CA, USA; 2 Experimental and Clinical Research Center, Max Delbrueck Center for Molecular Medicine, 13125 Berlin, Germany; 3 Clinic for Cardiology and Angiology, Charité University-Medicine Berlin, Campus Mitte, 10117 Berlin, Germany; 4 Laboratory of Anesthesiology & Critical Care Medicine, Translational Neuroscience Center, West China Hospital, Sichuan University, Chengdu, Sichuan 610041, China

## Abstract

Nonalcoholic fatty liver disease (NAFLD) is an increasing health problem worldwide, with genetic, epigenetic, and environmental components. Here, we describe the first example of NAFLD caused by genetic disruption of a mammalian potassium channel subunit. Mice with germline deletion of the KCNE2 potassium channel β subunit exhibited NAFLD as early as postnatal day 7. Using mouse genetics, histology, liver damage assays and transcriptomics we discovered that iron deficiency arising from KCNE2-dependent achlorhydria is a major factor in early-onset NAFLD in *Kcne2*^─/─^ mice, while two other KCNE2-dependent defects did not initiate NAFLD. The findings uncover a novel genetic basis for NAFLD and an unexpected potential factor in human KCNE2-associated cardiovascular pathologies, including atherosclerosis.

NAFLD is the predominant liver disorder in many developed and developing countries, affecting as much as a third of the United States population^1^. Characterized by abnormally high hepatic lipid accumulation, NAFLD is of particular importance because it can progress to more dangerous disorders including nonalcoholic steatohepatitis (NASH) and potentially fatal liver cirrhosis^2^. NAFLD is commonly associated with metabolic syndrome, hypercholesterolemia and hypertriglyceridemia, and is often observed in obese or diabetic individuals, those with poor eating habits, or people who have experienced rapid weight loss. In addition, people without these risk factors can also develop NAFLD, and the incidence and severity of the disease is influenced by a variety of genetic and epigenetic factors in addition to lifestyle and other environmental influences^1^.

Sequence variants in six genes have been both linked to human NAFLD and independently validated (for review see^1^). The I148M variant in *PNPLA3*, which encodes patatin-like phospholipase domain-containing protein 3, is the major recognized genetic basis for NAFLD in human populations^3^. When the PNPLA3 I148M human NAFLD-associated polymorphism (rs738409) is overexpressed in mice, it results in triacyglycerol (TAG) accumulation. Similar results are obtained by targeted hepatic overexpression of wild-type *PNPLA3* in mice, via increased TAG and fatty acid synthesis and impaired hydrolysis of TAG; a relative depletion of long-chain polyunsaturated forms of TAG was also observed^4^,^5^. The other five genes are *GCKR* (which regulates glucokinase activity and hepatic glucose intake)^6^; *PEMT*, a catalyst for phosphatidylcholine synthesis^7^; SOD2 (which clears mitochondrial reactive oxygen species and protects against cell death)^8^; *KLF6* (a transcription factor that influences fibrogenesis)^9^; and ATGR1 (angiotensin type 1 receptor)^10^. In addition to these genetic factors and metabolic syndrome, hepatic iron also influences lipid metabolism and hepatic steatosis. Iron overload can cause oxidative stress and lipid peroxidation, and can, for example, increase the formation of intracellular lipid droplets in liver cells *in vitro*. Conversely, iron deficiency has been shown to increase lipogenesis in rat liver, resulting in triglyceride accumulation and steatosis^11^. Thus, NAFLD is a common and highly complex pathological state affected by many different interacting factors that can potentially influence its onset and development into more severe diseases.

We previously found that targeted deletion of the *Kcne2* gene causes iron deficiency anemia, and also hypercholesterolemia^12^. KCNE2 is a potassium channel β subunit linked to cardiac arrhythmias and atherosclerosis^13^,^14^,^15^. The five-strong KCNE gene family comprises single transmembrane span proteins (KCNE subunits, also referred to as Mink-related peptides or MiRPs) that co-assemble with and alter the functional attributes of voltage-gated potassium (Kv) channel pore-forming (α) subunits^16^. Like other KCNE subunits, KCNE2 is widely expressed in a variety of tissues, and can promiscuously associate with several different Kv α subunits^17^.

Aside from its roles in cardiac myocytes, where KCNE2 regulates hERG, Kv4.2, Kv1.5 and Kv2.1 depending on the species^13^,^18^,^19^,^20^,^21^, KCNE2 also co-assembles with the KCNQ1 α subunit^22^. This complex is important for various epithelial tissues, including the stomach, thyroid and choroid plexus^18^,^19^,^23^,^24^. Importantly, *Kcne2*^─/─^ mice exhibit achlorhydria, because KCNQ1-KCNE2 channels are required for normal function of the parietal cell H^+^/K^+^-ATPase, and therefore gastric acid secretion^24^,^25^. *Kcne2* deletion results in mis-trafficking of KCNQ1 channels to the basolateral side of parietal cells, where they cannot fulfil their normal function, and ultimately leads to gastritis cystica profunda and gastric neoplasia^26^,^27^. Because the *Kcne2*-linked achlorhydria impairs iron uptake and causes iron deficiency anemia, a potential cause of abnormalities in hepatic lipid metabolism, here we investigated *Kcne2*-dependent hepatic lipid content and transcriptome remodeling, and discovered that *Kcne2* deletion causes NAFLD.

## Results and Discussion

**Figure 1:**
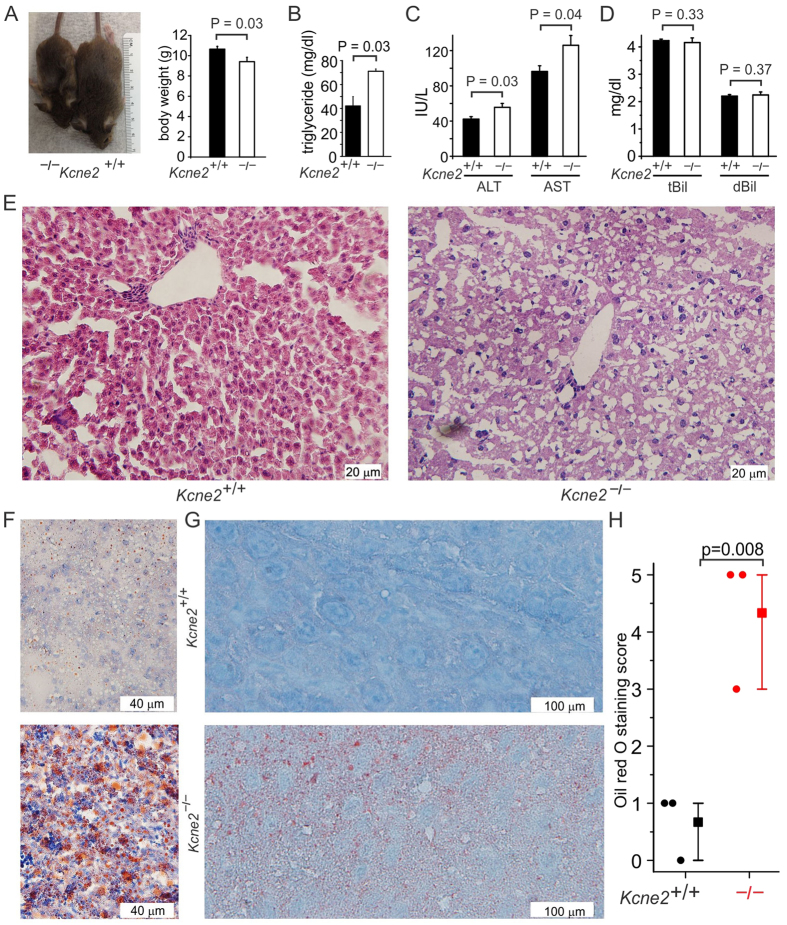
*Kcne2* deletion causes NAFLD. (**A**) Representative image (*left*) and mean body weight (*right*) of 3-week-old male *Kcne2*^+*/*+^ and *Kcne2*^*─/─*^ mice (*n* = 8). Statistical analysis was by 2-tailed student’s t-test. (**B**) Mean serum triglyceride concentration for normal diet-fed 3-week-old male *Kcne2*^+*/*+^ and *Kcne2*^*─/─*^ mice (*n* = 3 per group). Statistical analysis was by 2-tailed student’s t-test. (**C**) Mean serum ALT and AST concentrations for normal diet-fed 3-week-old *Kcne2*^+*/*+^ and *Kcne2*^*─/─*^mice (*n* = 6–7 per group). Statistical analysis was by 2-tailed student’s t-test. (**D**) Mean serum total (t) and direct (d) bilirubin (Bil) concentration for normal diet-fed 3-week-old male *Kcne2*^+*/*+^ and *Kcne2*^*─/─*^ mice (*n* = 5–7 per group). Statistical analysis was by 2-tailed student’s t-test. (**E**) Representative images of hematoxylin and eosin-stained liver left lobe sections from 3-week-old *Kcne2*^+*/*+^ and *Kcne2*^*─/─*^ mice (*n* = 3). (**F**) Representative images of oil red O stained liver left lobe sections from 1-week-old *Kcne2*^+*/*+^ and *Kcne2*^*─/─*^mice (*n* = 3). (**G**) Representative images of oil red O stained liver left lobe sections from 3-week-old *Kcne2*^+*/*+^ and *Kcne2*^*─/─*^mice (*n* = 3). (**H**) Oil red O stain blinded scores for images as in panel G (*n* = 3). A score of 5 indicates strongest staining.

Postnatal day 21 (P21) *Kcne2*^─/─^ pups exhibited lower bodyweight compared to wild-type (*Kcne2*^+/+^) counterparts (Fig. 1A), but had elevated serum triglycerides (Fig. 1B). Serum ALT and AST levels were also elevated in P21 *Kcne2*^─/─^ pups (Fig. 1C) whereas bilirubin was unchanged (Fig. 1D). These findings are consistent with early NAFLD, which was further explored using histology. Liver tissue from P21 *Kcne2*^─/─^ pups had a more vacuolated appearance than those from wild-type pups (Fig. 1E) and *Kcne2* deletion caused marked accumulation of lipid in both P7 (Fig. 1F) and P21 (Fig. 1G, H) pup livers, confirming that *Kcne2*^─/─^ pups had early-onset NAFLD.

**Figure 2:**
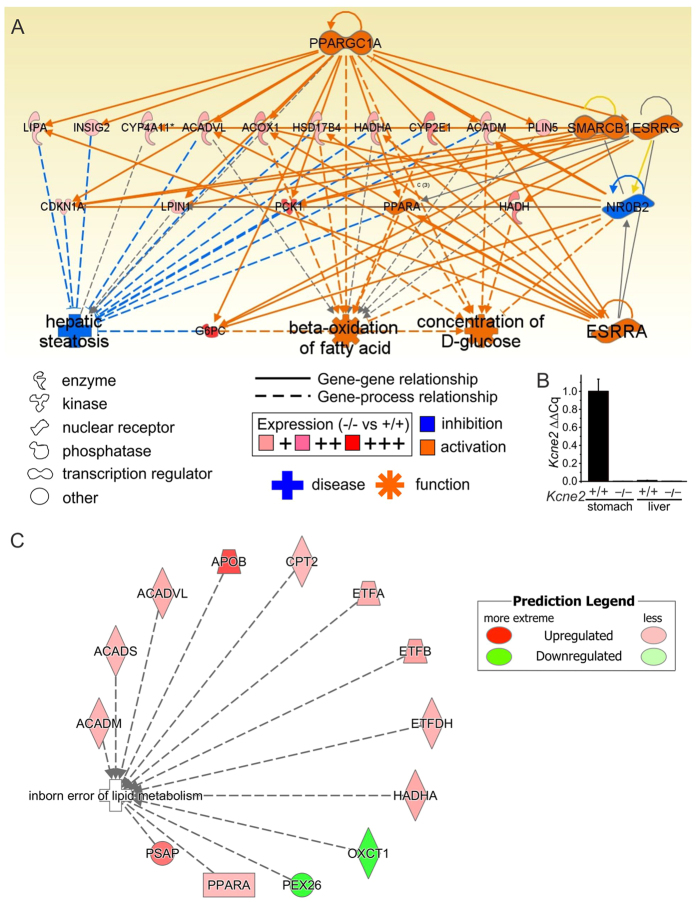
*Kcne2*-dependent changes in interacting hepatic gene networks. (**A**) The liver DEG interacting networks (after microarray transcriptome analysis of liver tissue from global *Kcne2*^─/─^ versus *Kcne2*^+/+^ mice, *n* = 8 mice per group) with the highest consistency score included beta-oxidation of fatty acids, glucose concentration and hepatic steatosis, with predicted hierarchical control by PPARGC1A (IPA software, Qiagen). (**B**) Real-time qPCR does not detect *Kcne2* transcript expression in *Kcne2*^+/+^ mouse liver. *Kcne2*^+/+^ stomach tissue was used as a positive control, *Kcne2*^*─/─*^tissue as a negative control; *n* = 3–4 mice per group, each quantified in triplicate. (**C**) The first-ranked disease process/biological function gene set altered by *Kcne2* deletion was *Inborn error of lipid metabolism*, identified by *Ingenuity* pathway analysis of hepatic transcriptome changes quantified by microarray analysis as in panel A (P = 5.1 × 10^−7^, *n* = 8 per genotype).

Microarray transcriptome analysis (Supplemental Spreadsheet 1) followed by regulator effect analysis (*Ingenuity Pathway Analysis*, Qiagen) of differentially expressed genes (DEGs) in livers of P21 *Kcne2*^─/─^ pups compared to *Kcne2*^+/+^ littermates identified the network with the highest consistency score as one including beta-oxidation of fatty acids, glucose concentration and hepatic steatosis, controlled by the transcriptional co-activator and regulator of genes important for energy metabolism, Peroxisome proliferator-activated receptor gamma co-activator 1-α (PGC-1α, encoded by PPARGC1A) (Fig. 2A). The specific transcriptional changes observed within functional gene networks in the liver were highly consistent with remodeling in response to development of NAFLD, e.g., increased beta-oxidation of fatty acids in response to the lipid accumulation, and increased glucose-6 phosphatase catalytic subunit (G6PC) expression (knockout of glucose-6 phosphatase causes hepatic steatosis in mice^28^). Thus, the associated transcriptome changes were likely not causing the NAFLD but were the result of remodeling in response to it, indicating that the *Kcne2*^─/─^ liver was responding (albeit insufficiently) to abnormally high lipid accumulation. These changes occurred despite the lack of *Kcne2* expression in *Kcne2*^+/+^ mouse liver (Fig. 2B), suggesting that *Kcne2*-dependent NAFLD arose via an initially extrahepatic defect.

Again employing an unbiased comparison of *Kcne2*^─/─^ versus *Kcne2*^+/+^ mouse liver transcriptomes from P21 pups, *Ingenuity* pathway analysis software identified *Inborn error of lipid metabolism* as the primary disease/biological function gene set exhibiting differential expression arising from *Kcne2* deletion (Fig. 2C). The gene within this set exhibiting the greatest magnitude change in hepatic expression was *apolipoprotein B* (*ApoB*), which was 3.2-fold upregulated in livers from *Kcne2*^─/─^ versus *Kcne2*^+/+^ mice (*n* = 8 per genotype, P < 0.05). This is of particular interest because ApoB is the predominant apolipoprotein of low and intermediate density lipoproteins and chylomicrons, with one ApoB100 molecule per hepatic-derived lipoprotein (ApoB100 being the full-length protein product of the *ApoB* gene and the only form of the protein synthesized in the liver). Hepatic ApoB expression is therefore a useful index of hepatic lipoprotein concentration, and its increase in response to *Kcne2* deletion is highly consistent with NAFLD. Furthermore, previous studies showed that the human ApoB/A1 ratio positively correlates (independent of other risk factors) with prevalence of NAFLD^29^. Our transcriptomic analysis indicates that the hepatic ApoB/A1 transcript ratio is increased 2.3-fold by *Kcne2* deletion (Supplemental Spreadsheet 1), again consistent with a diagnosis of NAFLD in *Kcne2*^─/─^ mice.

**Figure 3:**
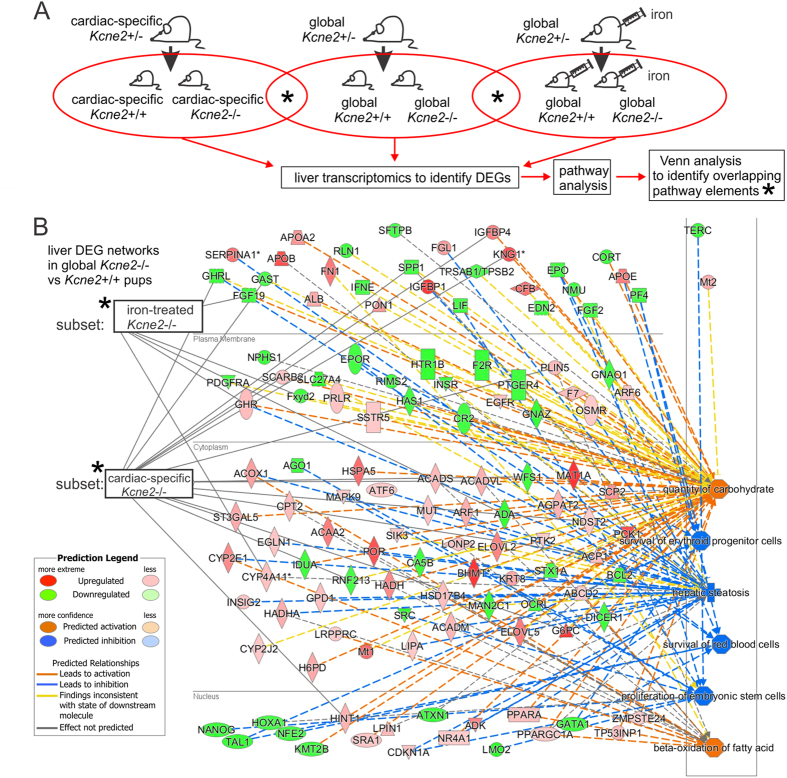
Iron supplementation prevents NAFLD-associated transcriptome changes in *Kcne2*^─/─^ mouse livers. (**A**) Schema showing genotypes, treatment and analyses for *Kcne2*-dependent hepatic transcriptome analysis. (**B**) DEG networks (IPA software, Qiagen) when comparing liver tissue harvested from 3-week-old global-knockout *Kcne2*^─/─^ versus *Kcne2*^+/+^ pups (organized by cellular compartment), after microarray transcriptome analysis (*n* = 8 mice per group). Red, upregulated; green, downregulated, in *Kcne2*^─/─^ versus *Kcne2*^+/+^ livers. Venn analysis revealed that iron supplementation from birth eliminated all but 5 transcript changes (subset: iron treated) and that cardiac-specific *Kcne2* deletion resulted in only 15 liver DEGs (subset: cardiac-specific *Kcne2*^─/─^) common to those altered by global *Kcne2* deletion.

*Kcne2* deletion-linked achlorhydria^24^ causes iron-deficiency anemia^12^,^30^, which can predispose to dyslipidemia and NAFLD^11^. Although data vary depending on the animal model studied, iron deficiency in rats, for example, has been reported to increase hepatic lipogenesis, causing steatosis; this may occur via increased *de novo* lipogenesis from glucose^31^. Here, to investigate the possible role of iron deficiency in *Kcne2* deletion-linked NAFLD, we initially utilized transcriptomic analysis in conjunction with iron supplementation. Non-treated P21 *Kcne2*^─/─^ livers exhibited extensive transcriptome remodeling indicative of NAFLD and anemia (Supplemental Spreadsheet 1). The 6 top-ranked DEG networks as identified by pathway analysis were: increased beta oxidation of fatty acids, elevated carbohydrates, hepatic steatosis, survival of erythroid progenitor cells and red blood cells, and proliferation of embryonic stem cells (Fig. 3). Strikingly, supplementation with injectable iron (iron dextran) eliminated the differences in concerted gene expression caused by *Kcne2* deletion that are associated with anemia (demonstrating that the iron supplementation we employed was effective in restoring iron levels and preventing anemia) and also the gene expression changes associated with NAFLD. Thus, only 5 of the 116 DEGs in the top 6 DEG networks were still differentially expressed in *Kcne2*^─/─^ livers after iron treatment (Fig. 3; Supplemental Spreadsheet 2).

**Figure 4:**
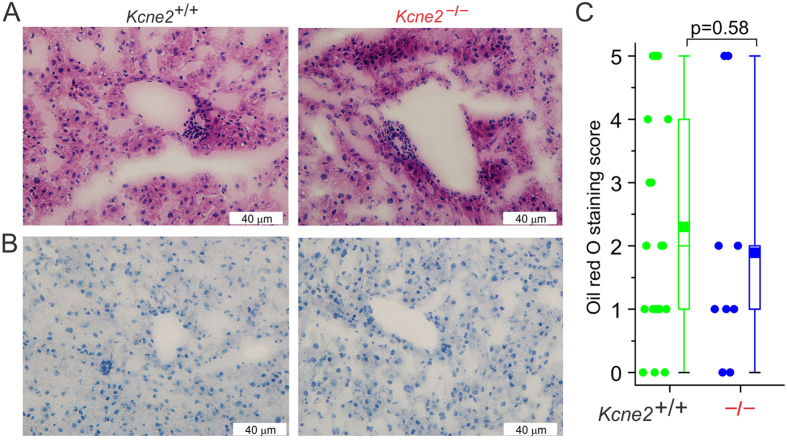
Iron supplementation prevents *Kcne2* deletion-associated NAFLD. (**A**) Representative images of hematoxylin and eosin-stained liver left-lobe sections from iron dextran-treated P21 global *Kcne2*^─/─^ versus *Kcne2*^+/+^ mice (*n* = 9–16). (**B**) Representative images of oil-red-O-stained liver left-lobe sections from iron dextran-treated P21 global *Kcne2*^─/─^ versus *Kcne2*^+/+^ mice (*n* = 9–16). (**C**) Oil red O stain blinded scores for images as in panel B (*n* = 9–16). A score of 5 indicates strongest staining.

Consistent with the finding that iron supplementation prevented the transcriptome changes associated with NAFLD in *Kcne2*^─/─^ pups, iron supplementation also prevented excessive vacuolation in the liver and eliminated *Kcne2*-dependent differences in hepatic lipid accumulation in the liver (Fig. 4A–C). A comparison of hepatic oil red O staining in iron-treated versus non-treated *Kcne2*^─/─^ pups indicated successful alleviation of hepatic steatosis by iron supplementation (one-way ANOVA, p = 0.03). Thus, iron deficiency is a major factor in *Kcne2*-dependent, early-onset NAFLD. Note that the moderate *increase* in hepatic lipids of *Kcne2*^+/+^ pups treated with iron likely arose from iron overload which, as with iron deficiency, can also cause hepatic steatosis^11^.

To increase confidence that iron deficiency played the major role in *Kcne2*-dependent NAFLD, we examined other potential causes. *Kcne2* deletion also causes cardiac dysfunction, which can lead to right-heart failure and associated liver fibrosis^12^,^19^,^20^. Although the livers studied here were from global *Kcne2*^─/─^ pups at P21, at which age they do not show signs of heart failure^12^,^19^,^20^, we next examined livers isolated from mice with a cardiac-specific *Kcne2* deletion, to rule out a direct role for cardiac dysfunction in *Kcne2*-dependent NAFLD. Accordingly, only 15/116 DEGs in the top 6 DEG networks identified in global *Kcne2*^─/─^ mouse livers were also differentially expressed in cardiac-specific *Kcne2*^─/─^ mouse livers, strongly suggesting against a cardiac role in *Kcne2*^─/─^ NAFLD initiation (Fig. 3; Supplemental Spreadsheet 3).

**Figure 5:**
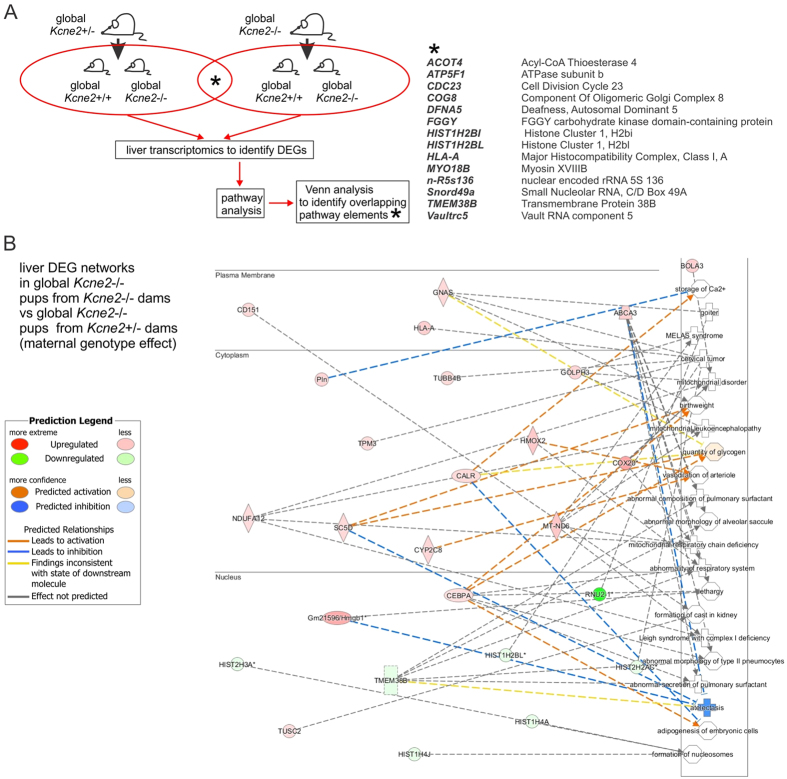
NAFLD in P21 *Kcne2*^─/─^ pups is not altered by maternal genotype. (**A**) *Left*, schema showing genotypes, treatment and analyses for effects of maternal genotype on *Kcne2*-dependent hepatic steatosis study. *Right*, the 14 DEGs identified when comparing livers of *Kcne2*^─/─^ pups versus those of *Kcne2*^+/+^ pups (Fig. 3) that were also differentially expressed in *Kcne2*^─/─^ pups from *Kcne2*^─/─^ dams versus those from *Kcne2*^+/─^ dams; none of these were within the 6 identified anemia/NAFLD networks from Fig. 3. **(B**) DEG networks (IPA software, Qiagen) when comparing liver tissue harvested from 3-week-old global-knockout *Kcne2*^─/─^ pups from *Kcne2*^─/─^ dams versus *Kcne2*^─/─^ pups from *Kcne2*^+/─^ dams (organized by cellular compartment), after microarray transcriptome analysis (*n* = 8 mice per group). Red, upregulated; green, downregulated, in *Kcne2*^─/─^ versus *Kcne2*^+/+^ livers.

*Kcne2* deletion also results in hypothyroidism because KCNQ1-KCNE2 channels facilitate thyroid iodide uptake by the sodium iodide symporter^19^,^32^. Pups of *Kcne2*^─/─^ dams are hypothyroid regardless of their own genotype because they rely on milk for iodide and/or thyroid hormones, whereas *Kcne2*^─/─^ mice bred from *Kcne2*^+/─^ dams do not exhibit signs of hypothyroidism until adulthood^19^. However, because hypothyroidism is a risk factor for NAFLD and even upper-normal levels of TSH associate with human NAFLD^33^, and because we previously observed findings suggestive of liver fibrosis in hypothyroid *Kcne2*^─/─^ mice^19^, here we nevertheless examined this possibility, by comparing livers of P21 *Kcne2*^─/─^ pups bred from *Kcne2*^+/─^ versus *Kcne2*^─/─^ dams. Only 14 DEGs identified when comparing livers of *Kcne2*^─/─^ pups versus those of *Kcne2*^+/+^ pups (Fig. 3) were also identified as being differentially expressed in *Kcne2*^─/─^ pups from *Kcne2*^─/─^ dams versus those from *Kcne2*^+/─^ dams (Fig. 5A), and none of these were within the 6 identified anemia/NAFLD networks (Fig. 3). Furthermore, network analysis of DEGs in livers of *Kcne2*^─/─^ pups from *Kcne2*^─/─^ dams versus those from *Kcne2*^+/─^ dams revealed less-populated networks, spanning a range of physiological processes and not biased toward NAFLD (Fig. 5B). *Kcne2*-dependent NAFLD in pups from heterozygous dams, as we used in the current study, is therefore *not* initiated by hypothyroidism.

Taken together, our data support a primary role for iron deficiency, but not hypothyroidism or heart failure, in initiating *Kcne2*-dependent NAFLD. Similarly, iron deficiency can predispose to dyslipidemia and NAFLD in human populations^11^, both of which are risk factors for atherosclerosis^34^, as are certain human KCNE2 polymorphisms^3^.

**Figure 6:**
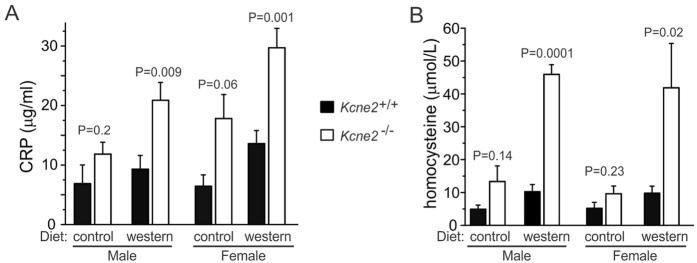
*Kcne2* deletion causes elevated serum CRP and homocysteine. (**A**) Mean serum C-reactive protein (CRP) concentration for 6–9 month-old *Kcne2*^+*/*+^ and *Kcne2*^*─/─*^mice. *n* = 7–10, male control diet; *n* = 8, male western diet; *n* = 4–7, female control diet; *n* = 8, female western diet; P values are for 2-tailed, unpaired t-tests for inter-genotype comparisons within equivalent sex and diet groups. (**B**) Mean serum homocysteine concentration for 6–9-month-old *Kcne2*^+*/*+^ and *Kcne2*^*─/─*^mice. *n* = 4, male control diet; 4, male western diet; 3–4, female control diet; 3–5, female western diet; p values are for 2-tailed, unpaired t-tests for inter-genotype comparisons within equivalent sex and diet groups.

In addition, we found that serum C-reactive protein (CRP), an atherosclerotic biomarker released by the liver in response to inflammation, was elevated in *Kcne2*^─/─^ mice - most prominently in western diet-fed females (Fig. 6A). This correlates with recently-discovered atherosclerotic predisposition in *Kcne2*^─/─^ mice^15^ and is also consistent with presence of NAFLD, although elevated CRP is not a specific biomarker for NAFLD^35^. Achlorhydria also causes hyperhomocysteinemia, another atherosclerosis risk factor^36^; accordingly, *Kcne2* deletion increased serum homocysteine (Fig. 6B), providing another possible mechanistic link between achlorhydria (impairing vitamin B absorption, causing hyperhomocysteinemia) and atherosclerosis in *Kcne2*^─/─^ mice. Importantly, hyperhomocysteinemia has also been positively correlated with NAFLD in human populations, and in mouse models has been suggested to lead to hepatic steatosis via abnormal lipid metabolism^37^. Thus, although iron supplementation prevents hepatic steatosis in *Kcne2*^─/─^ mice, it is possible that their hyperhomocysteinemia also contributes or predisposes to NAFLD in the setting of anemia (perhaps in a double-hit scenario).

In conclusion, our data support a substantial role for *Kcne2*-linked iron deficiency in the development of NAFLD in mice. Future studies will determine if this novel genetic link is recapitulated in humans, and investigate potential interactions between KCNE2-associated atherosclerosis, dyslipidemia, NAFLD, and diet-dependent cardiac arrhythmogenesis and sudden death^15^.

## Methods

### Generation of mice and study protocol

The *Kcne2*^─/─^ mouse line was generated as we previously described^24^, and mice used in this study were bred by crossing *Kcne2*^+*/*─^ mice which had been backcrossed >10 times into the C57BL/6 strain. After being genotyped and weaned at 3 weeks of age, mice pups were assigned to, and maintained on, either a control diet (2020X, Harlan, 16% kcal from fat; 19.1% protein, 2.7% crude fiber, 12.3% neutral detergent fiber and 0% cholesterol) or western diet (TD.88137, Harlan, 42% kcal from fat, >60% of which is saturated; 34% sucrose; 0.2% cholesterol). Cardiac-specific *Kcne2*^─/─^ mice, used as a control in the liver analyses, were generated using a mouse line expressing Cre-recombinase under the control of the αMHC (alpha myosin heavy chain) promoter; a full phenotypic description of this mouse line will appear in a separate, future study. Mouse tissue and blood serum were then collected for further analysis or stored at −80 °C.

### Whole-transcript Microarray analysis

Mice were euthanized, and then tissue was harvested and preserved in RNA*later (*Invitrogen) until use. Total RNA was collected from the liver, reverse-transcribed into cDNA and analyzed by “whole-transcript transcriptomics” using the GeneAtlas microarray system (Affymetrix) and manufacturer’s protocols. MoGene 1.1 ST array strips (Affymetrix) were used to hybridize to newly synthesized sscDNA. Each array comprised 770,317 distinct 25-mer probes to probe an estimated 28,853 transcripts, with a median 27 probes per gene. Gene expression changes associated with *Kcne2* deletion were analyzed using Ingenuity Pathway Analysis (Qiagen) to identify biological networks, pathways, processes and diseases that were most highly represented in the differentially expressed gene (DEGs) identified. Expression changes of ≥1.5 fold and p < 0.05 were included in the analysis.

### RNA isolation and Real-Time qPCR

Mice were euthanized by CO_2_ asphyxiation. Gastric fundus tissue was harvested and washed with PBS; livers were harvested, washed and perfused through left ventricle with PBS + heparin, then all tissue either processed or stored at −80 °C until use. RNA was extracted using 1 ml of Trizol (Invitrogen) per 100 mg of tissue and purified using the RNeasy Mini Kit (Qiagen) according to the manufacturer’s protocol. RNA samples with A_260_/A_280_ absorbance ratios between 2.00–2.20 were used for further synthesis. 500 ng to 1 μg of RNA was used for cDNA synthesis (Qiagen’s Quantitect Reverse Transcriptase) and stored at −20 °C until use. Primer pairs for target gene *Kcne2* (NCBI Gene ID: 246133) and *Gapdh* (NCBI Gene ID: 14433) produced amplicons of 175 bp and 123 bp respectively. The qPCR primer sequences were as follows:

*Kcne2*, forward 5′-CACATTAGCCAATTTGACCCAG-3′, and reverse 5′-GAACATGCCGATCATCACCAT-3′; *Gapdh,* forward 5′-AGGTCGGTGTGAACGGATTTG-3′; and reverse 5′-TGTAGACCATGTAGTTGAGGTCA-3. Primers (0.05 μm synthesis scale, HPLC purified) were acquired from Sigma. Real-time qPCR analysis was performed using the CFX Connect System, iTaq Universal SYBR Green Supermix (BioRad) and 96-well clear plates. Thermocycling parameters were set according to manufacturer’s protocol for iTaq. Samples were run in triplicate as a quality control measure and triplicates with a standard deviation of 0.6 or higher were repeated. Melting curves were assessed for verification of a single product. ∆∆Cq values were normalized to those obtained for the *Kcne2*^+*/*+^ stomach tissue.

### Iron supplementation study

To determine the potential beneficial effects of alleviating iron-deficiency anemia, post-partum dams were first intraperitoneally (IP) injected with iron dextran (25 mg/kg) or vehicle control (saline) on the day their pups were born. Mouse pups were then injected with iron dextran (12.5 mg/kg) or vehicle control (saline) at P7 and P14. Whole livers and blood serum were then harvested for analysis at P21. Liver section oil red O staining was performed by UC Irvine pathology core facility, and then the extent of staining was quantified by a scorer blinded to genotype, treatment, and hypothesis. Representative images were then chosen based on the mean score for each group.

### Blood analysis

To quantify triglycerides, serum was collected after euthanasia from 3-week-old male mouse pups and then analyzed using a glycerol oxidation-based colorimetric assay (Abcam, United Kingdom). CRP and homocysteine were quantified in serum collected after sacrificing 6–9-month-old mice, using ELISA (R&D systems, MN) and the Mouse Homocysteine Assay kit (quantifying hydrogen sulfide resulting from degradation of homocysteine by homocysteinase) (Crystal Chem, IL, USA), respectively. Alanine transaminase (ALT), aspartate transaminase (AST), and total and direct (conjugated) bilirubin concentrations in P21 mouse serum were quantified using a Mindray BS-120 Chemistry Analyzer (Mindray Medical Corporation, Shenzhen, China).

### Statistical analysis

Statistical analyses (student’s t-test or ANOVA, as indicated in the figure legends) were performed assuming statistical significance with p values <0.05.

### Study approval

All mice were housed in pathogen-free facilities and the study was approved by the Animal Care and Use Committee at University of California, Irvine. Studies were performed during the light cycle and were carried out in strict accordance with the recommendations in the Guide for the Care and Use of Laboratory Animals of the National Institutes of Health.

## Additional Information

**How to cite this article**: Lee, S. M. *et al*. *Kcne2* deletion causes early-onset nonalcoholic fatty liver disease via iron deficiency anemia. *Sci. Rep.* **6**, 23118; doi: 10.1038/srep23118 (2016).

## Supplementary Material

Supplementary Spreadsheet 1

Supplementary Spreadsheet 2

Supplementary Spreadsheet 3
